# Microwave-Heating-Assisted Synthesis of Ultrathin and Ultralong Hydroxyapatite Nanowires Using Biogenic Creatine Phosphate and Their Derived Flexible Bio-Paper with Drug Delivery Function

**DOI:** 10.3390/molecules30050996

**Published:** 2025-02-21

**Authors:** Yu Zhang, Ying-Jie Zhu, Si-Yi Li, Li-Ying Dong, Han-Ping Yu

**Affiliations:** 1 State Key Laboratory of High Performance Ceramics and Superfine Microstructure, Shanghai Institute of Ceramics, Chinese Academy of Sciences, Shanghai 200050, China; zgzyaizg@163.com (Y.Z.); lain9221@163.com (S.-Y.L.); lydong@mail.sic.ac.cn (L.-Y.D.); 2 Center of Materials Science and Optoelectronics Engineering, University of Chinese Academy of Sciences, Beijing 100049, China

**Keywords:** hydroxyapatite, nanowire, microwave, hydrothermal, creatine phosphate

## Abstract

With an ultrahigh aspect ratio and a similar chemical composition to the biomineral in bone and tooth, ultralong hydroxyapatite nanowires (UHAPNWs) exhibit a meritorious combination of high flexibility, excellent mechanical performance, high biocompatibility, and bioactivity. Despite these exciting merits, the rapid and green synthesis of UHAPNWs remains challenging. In this work, we have developed an environment-friendly, rapid, and highly efficient synthesis of ultrathin UHAPNWs by the microwave-assisted calcium oleate precursor hydrothermal method using biogenic creatine phosphate as the bio-phosphorus source. Owing to the controllable hydrolysis of bio-phosphorus-containing creatine phosphate and the highly efficient heating of microwave irradiation, ultrathin UHAPNWs with a homogeneous morphology of several nanometers in diameter (single nanowire), several hundred micrometers in length, and ultrahigh aspect ratios (>10,000) can be rapidly synthesized within 60 min. This effectively shortens the synthesis time by about two orders of magnitude compared with the traditional hydrothermal method. Furthermore, ultrathin UHAPNWs are decorated in situ with bioactive creatine and self-assembled into nanowire bundles along their longitudinal direction at the nanoscale. In addition, ultrathin UHAPNWs exhibit a relatively high specific surface area of 84.30 m^2^ g^–1^ and high ibuprofen drug loading capacity. The flexible bio-paper constructed from interwoven ibuprofen-loaded ultrathin UHAPNWs can sustainably deliver ibuprofen in phosphate-buffered saline, which is promising for various biomedical applications such as tissue regeneration with anti-inflammatory and analgesic functions.

## 1. Introduction

With the rapid and energetic development of nanotechnology in recent decades, various kinds of nanomaterials have been created for widespread applications [1,2,3,4]. Nanowires, in particular, with their merits of anisotropy, superior mechanical properties, weaving ability, and high surface-to-volume ratio, have attracted tremendous attention in recent years [5,6]. Nanowires and their assembled materials usually exhibit very different physical and chemical properties from their monolithic bulk analogues [7,8]. For example, many ceramic nanowires have been found to be more flexible than traditional ceramic pieces [9,10,11,12], and they can be woven into a variety of porous scaffolds for tissue regeneration [13,14,15,16]. Moreover, nanowires can provide abundant surface-active points for high drug loading capacity and controlled drug release [17,18].

Hydroxyapatite (HAP, Ca_10_(PO_4_)_6_(OH)_2_) is a popular inorganic biomaterial for bone tissue engineering, tooth repair, and drug delivery [19,20,21]. HAP nanowires (HAPNWs) show outstanding properties, such as high mechanical properties, biocompatibility, bioactivity, osteo-conductivity, and osteo-induction, owing to their compositional similarity to the inorganic mineral in human bones and teeth [22,23]. It is well-accepted that high aspect ratio HAP nanostructures encourage rich functions [24]. In recent years, the authors’ research group has developed a kind of ultralong HAP nanowire (UHAPNW) with ultrahigh aspect ratios (>10,000), about 10 nm in diameter, and several hundred micrometers in length [25,26,27]. In contrast to the traditional brittle HAP ceramic materials, UHAPNWs exhibit high flexibility. UHAPNWs can be twisted and interwoven owing to their extremely high aspect ratios, and they can assemble into various flexible functional materials with the networked structure [26,27]. Moreover, UHAPNWs show a high surface-to-volume ratio and significant interface phenomena, providing abundant sites for the attachment of drug molecules, metal ions, and functional groups [25,26,27,28].

**Scheme 1:**
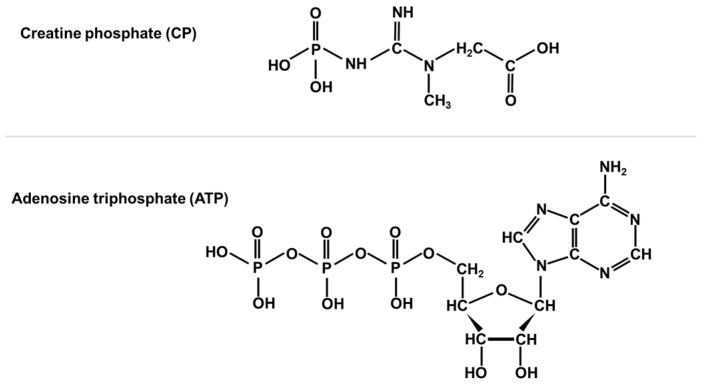
The chemical structures of creatine phosphate and adenosine triphosphate (ATP).

The traditional synthesis of HAP materials is still facing the challenges of uncontrollable formation of calcium phosphate nuclei and lengthy reaction times [29,30,31]. In our previous work, we developed a microwave-assisted calcium oleate precursor–bio-phosphorus hydrothermal method, which is promising for the preparation of UHAPNWs with uniform morphology, high mechanical flexibility, and high bioactivity [32]. Moreover, owing to the controlled hydrolysis of phosphorus-containing biomolecules as well as the rapid and uniform heating by microwaves [33,34], the nucleation and growth of crystals can be regulated, resulting in HAP nanowires with small diameters and high aspect ratios. Unfortunately, until now, only adenosine triphosphate (ATP) has been explored for the synthesis of UHAPNWs. The chemical structure of ATP is shown in Scheme 1. More potential phosphorus-containing biomolecules need to be explored, yet this is challenging. Unlike the case of inorganic phosphates as the phosphorus source, the complexity of the structure and hydrolysis process of phosphorus-containing biomolecules may prohibit the satisfactory preparation of high-quality UHAPNWs.

Creatine phosphate (phosphocreatine) is a high-energy phosphate compound commonly found in muscle or other excitable tissues [35,36]. The chemical structure of creatine phosphate is shown in Scheme 1. It is a storage form of high-energy phosphate groups. When organisms consume ATP and produce adenosine diphosphate, creatine phosphate can rapidly release high-energy phosphate groups into adenosine diphosphate under enzymatic action, so that ATP can be rapidly restored to the normal high supply levels [37]. In the past, studies focused on the in vitro hydrolysis of creatine phosphate and its mechanisms of interconversion with ATP and creatine in order to understand its role in influencing the body during exercise [38]. Because of its close association with high-energy phosphate compounds, creatine phosphate is often used as a reference for testing the metabolic performance of muscle [39,40,41]. In recent years, creatine phosphate has been explored for the synthesis of nanostructured materials. For instance, Cao et al. reported a microwave solvothermal method for the preparation of hollow hierarchical mesoporous structured hydroxyapatite microflowers with a high drug loading capacity using creatine phosphate as the phosphorus source and template [42]. Qi et al. used creatine phosphate to synthesize magnesium phosphate pentahydrate nanosheets with pH-responsive solubility, which is promising for use as pH-responsive nanocarriers for drug and gene delivery [43]. However, neither of them successfully prepared UHAPNWs using creatine phosphate as the bio-phosphorus source. We speculate that creatine phosphate may be used as the bio-phosphorus source to synthesize UHAPNWs like ATP.

In this work, we successfully prepared UHAPNWs with ultrathin diameters (only several nanometers for a single nanowire) using creatine phosphate as the bio-phosphorus source and calcium oleate as the precursor by the microwave-assisted calcium oleate precursor hydrothermal method. The optimized parameters for the synthesis of ultrathin UHAPNWs were investigated. The as-prepared ultrathin UHAPNWs have ultrasmall diameters (only several nanometers for a single nanowire), ultralong lengths (hundreds of micrometers), and ultrahigh aspect ratios (>10,000), and can self-assemble along their longitudinal direction to form nanowire bundles with a high BET specific surface area (84.30 m^2^ g^−1^). Moreover, the as-prepared ultrathin UHAPNWs show a high ibuprofen loading capacity (0.33 g drug per gram of nanocarrier). After loading ibuprofen, ultrathin UHAPNWs still exhibit a high specific surface area of 79.21 m^2^ g^−1^. The ibuprofen-loaded ultrathin UHAPNWs can be further interwoven into flexible and porous bio-paper, and it can continuously release the drug in phosphate-buffered saline (PBS), which is promising for various biomedical applications such as inflammatory treatment and promoting tissue regeneration.

## 2. Results and Discussion

### 2.1. Characterization of Ultrathin UHAPNWs

**Figure 1:**
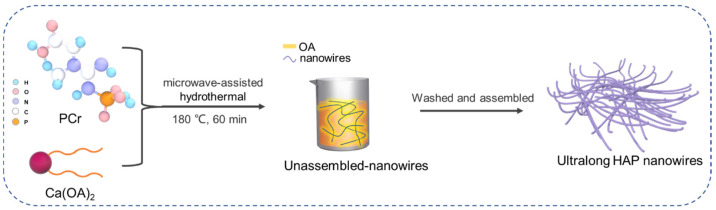
Schematic diagram of the rapid synthesis of ultrathin UHAPNWs by the microwave-assisted calcium oleate precursor hydrothermal method using sodium creatine phosphate as the bio-phosphorus source, and the self-assembly of ultrathin UHAPNWs into ultralong nanowire bundles.

In this work, we chose sodium creatine phosphate tetrahydrate as the bio-phosphorus source and calcium oleate as the precursor, and used microwave-assisted hydrothermal heating to rapidly synthesize ultrathin UHAPNWs. The synthetic process of ultrathin UHAPNWs is demonstrated in Figure 1. As shown in the schematic diagram, calcium ions are combined with oleate groups to form calcium oleate precursor. It is known that the phosphate groups are fixed within creatine phosphate biomolecules [44,45]. In the initial reaction system, there are no free PO_4_^3−^ ions because creatine phosphate molecules are not hydrolyzed to release free PO_4_^3−^ ions, which can prevent the premature formation of calcium phosphate nuclei and uncontrollable crystal growth. During microwave heating, the temperature of the reaction system rises rapidly, and creatine phosphate molecules hydrolyze and release free PO_4_^3−^ ions to supply the phosphorus source for the formation of ultrathin UHAPNWs. Moreover, microwave-assisted heating shows the advantages of rapid volumetric heating, higher efficiency and reaction rates, shorter reaction times, and lower energy consumption compared with the conventional heating methods [34,46,47,48], which is beneficial for controlling the hydrolysis of creatine phosphate molecules as well as for the environmentally friendly rapid synthesis of homogeneous and well-crystallized ultrathin UHAPNWs. In our previous review article [34], we discussed the comparison of energy consumption between microwave heating and conventional heating, confirming that the energy consumption of microwave heating is reduced significantly compared with conventional heating methods. In addition, in our previous studies, the microwave heating time needed for the synthesis of UHAPNWs was much shorter (less than 1 h) than the conventional heating methods (usually about 24 h or longer), and the microwave heating time needed for the synthesis of UHAPNWs decreased by about two orders of magnitude, further verifying that the energy consumption by microwave heating can be reduced significantly compared with conventional heating methods [25,28,32,47]. Microwave heating can significantly enhance the chemical reaction rate, selectivity, and yield of the product, and microwave heating synthesis can exhibit order-of-magnitude enhancements in the reaction rate as compared with the conventional heating synthesis. Due to the merits of microwave dielectric volumetric heating for rapid temperature ramping of the reaction system, the conventional heating system needs more than six times the energy consumption for the synthesis of cubic BaTiO_3_ nanoparticles compared with that of the microwave heating system [34,49]. As a result, the microwave heating calcium oleate precursor hydrothermal method provides a promising pathway to significantly enhance the efficiency and reduce the energy consumption for the rapid synthesis of UHAPNWs.

**Figure 2:**
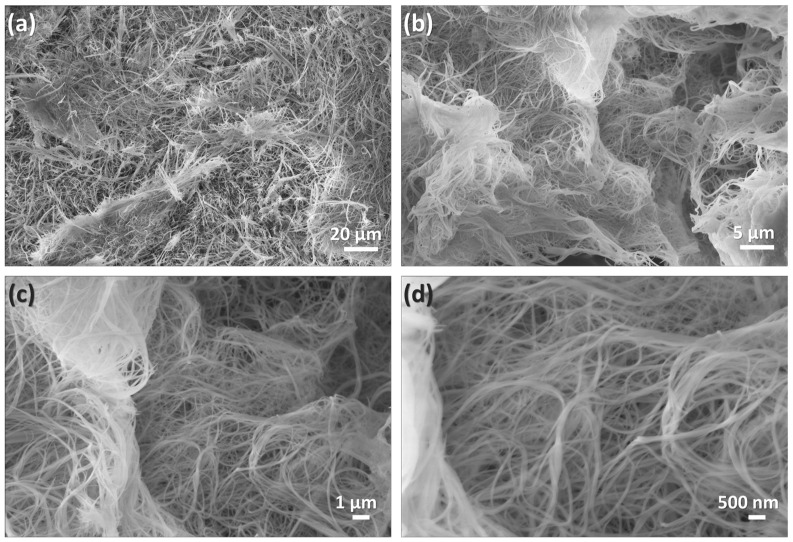
SEM images with different magnifications (**a**–**d**) of UHAPNWs prepared by the microwave-assisted calcium oleate precursor hydrothermal method using creatine phosphate as the bio-phosphorus source at 180 °C for 60 min.

Figure 2 shows the SEM images of the ultrathin UHAPNWs synthesized by the microwave-assisted calcium oleate precursor hydrothermal method using creatine phosphate as the bio-phosphorus source, and the reaction time and temperature are 60 min and 180 °C, respectively. As expected, ultrathin UHAPNWs can be successfully prepared by the microwave-assisted calcium oleate precursor hydrothermal method using creatine phosphate as the bio-phosphorus source. The as-prepared ultrathin UHAPNWs are morphologically homogeneous, and their diameters are dozens of nanometers, and their lengths can reach hundreds of micrometers. In addition, ultrathin UHAPNWs can self-assemble along their longitudinal direction to form nanowire bundles with larger diameters. Ultrathin UHAPNWs are highly flexible and can be bent at large angles without fracture. Due to the high aspect ratios and high flexibility of ultrathin UHAPNWs, they tend to interweave into a porous nanowire network.

**Figure 3:**
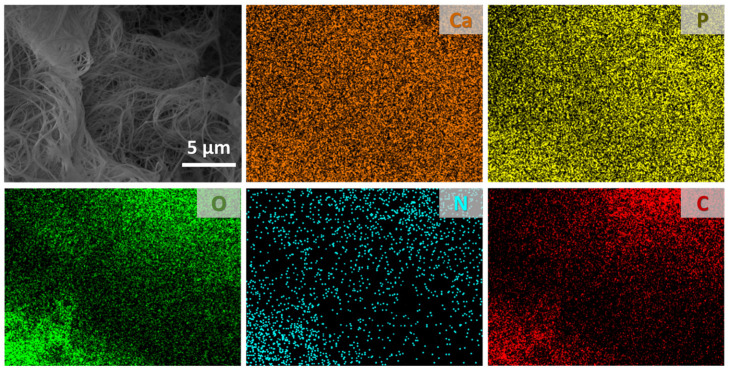
EDS elemental mapping of the ultrathin UHAPNWs prepared by the microwave-assisted calcium oleate precursor hydrothermal method using creatine phosphate as the bio-phosphorus source at 180 °C for 60 min.

Energy dispersive spectroscopy (EDS) elemental mapping results (Figure 3) indicate that the as-prepared ultrathin UHAPNWs contain Ca, P, O, N, and C elements. The Ca, P, and O elements originate from ultrathin UHAPNWs. Both the N and C elements are from the components of creatine phosphate, suggesting the presence of creatine phosphate molecules adsorbed on the surface of ultrathin UHAPNWs.

**Figure 4:**
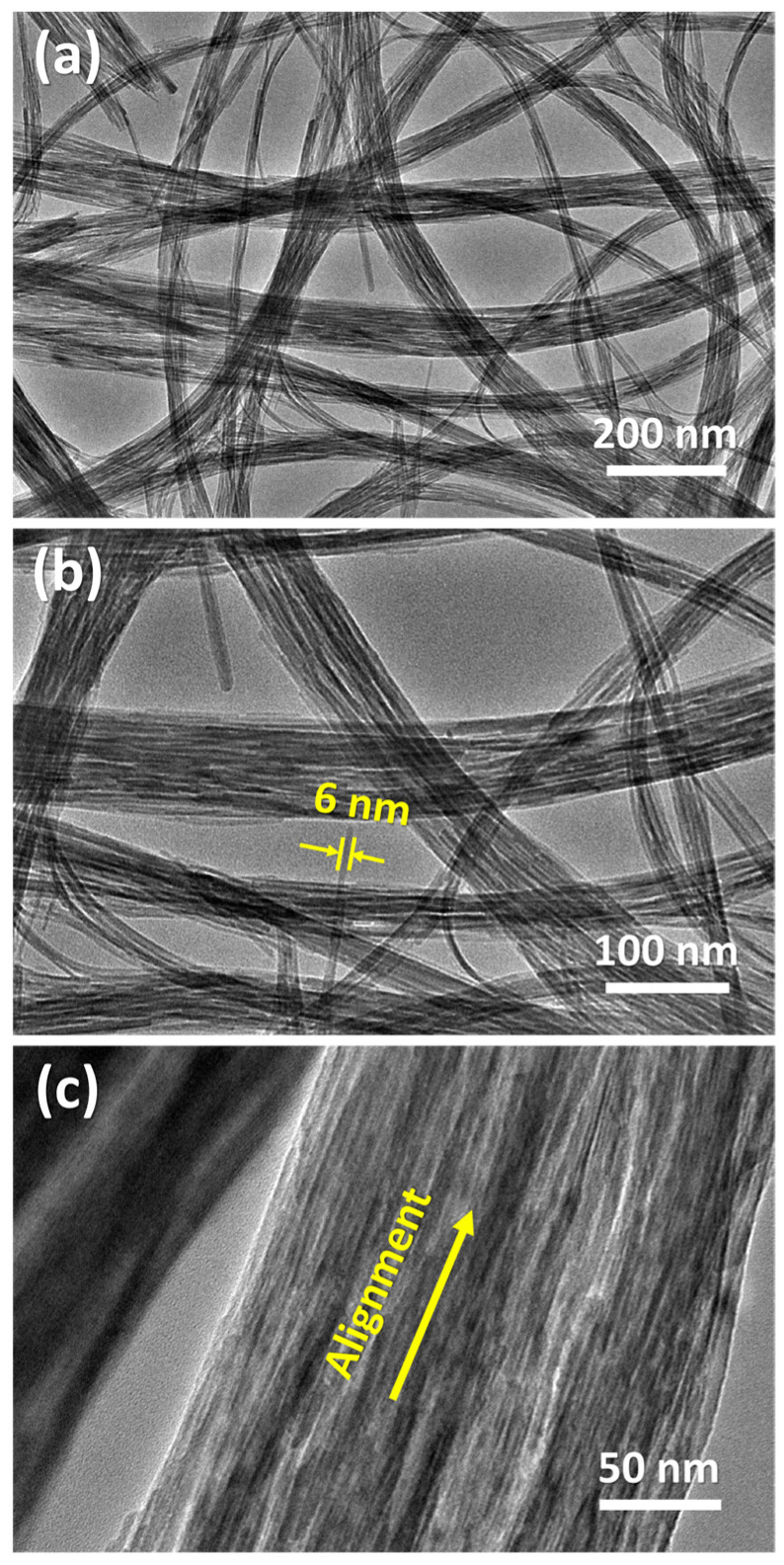
TEM images of ultrathin UHAPNWs and nanowire bundles with high flexibility (**a**), ultrasmall nanowire diameter (**b**), and nanoscale self-alignment (**c**) prepared by the microwave-assisted calcium oleate precursor hydrothermal method using creatine phosphate as the bio-phosphorus source at 180 °C for 60 min.

Higher-resolution structural analysis of the as-prepared ultrathin UHAPNWs was performed by TEM, and the results are shown in Figure 4. One can see that ultrathin UHAPNWs are self-assembled along their longitudinal direction to form nanowire bundles with diameters of 10 to ~100 nanometers (Figure 4a). The diameter of a single ultralong HAP nanowire is only ~6 nm (Figure 4b), which is lower than those of UHAPNWs prepared using inorganic phosphate salts as the phosphorus source, and consistent with those of UHAPNWs synthesized using ATP as the bio-phosphorus source in our previous work [32]. This suggests that the combination of rapid microwave heating and the use of phosphorus-containing biomolecules as the bio-phosphorus source can rapidly synthesize UHAPNWs with ultrasmall diameters compared with those prepared by the conventional heating methods using inorganic phosphates. It is calculated that the aspect ratios of the as-prepared ultrathin UHAPNWs are more than 10,000. The TEM image of the nanowire bundles clearly reveals that ultrathin UHAPNWs self-align along their longitudinal direction at the nanoscale (Figure 4c). Such nanoscale ordered structures are beneficial to the superior microscopic flexibility of the ultralong HAP nanowire bundles [50].

### 2.2. Effects of Experimental Parameters on the Formation of Ultrathin UHAPNWs

**Figure 5:**
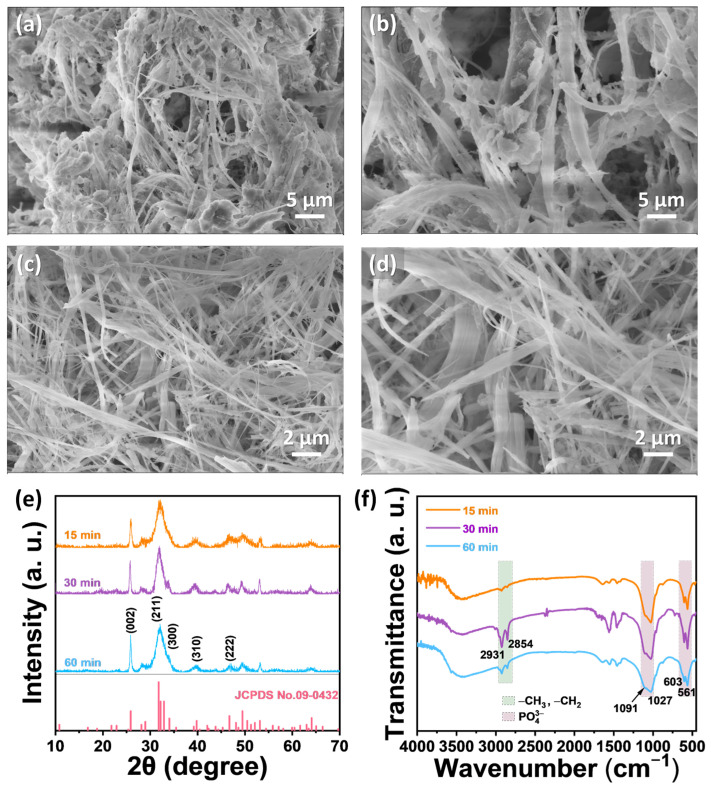
Evaluation of the influence of the reaction time on the formation of the product prepared by the microwave-assisted calcium oleate precursor hydrothermal method using creatine phosphate as the bio-phosphorus source at 180 °C. (**a**–**d**) SEM images of the products prepared for different reaction times: (**a**,**b**) 15 min; (**c**,**d**) 30 min. (**e**) XRD patterns. (**f**) FTIR spectra.

We explored the influence of the reaction time on the formation of ultrathin UHAPNWs prepared by the microwave-assisted calcium oleate precursor hydrothermal method using creatine phosphate as the bio-phosphorus source at 180 °C. As shown in Figure 5, one-dimensional (1-D) wire-like structures are formed for a reaction time of 15 min, accompanying many irregular-shaped particles (Figure 5a,b). When the microwave heating time increases to 30 min, the particles disappear and HAP nanowires are obtained. However, the as-prepared HAP nanowires show significantly heterogeneous sizes, and some microfibers with diameters of several micrometers can be observed (Figure 5c,d). As discussed above, when the microwave heating time further increases to 60 min, well-defined ultrathin UHAPNWs with a uniform morphology, ultrasmall diameters, ultralong lengths, ultrahigh aspect ratios, and high flexibility are obtained (Figure 2). In contrast to the traditional hydrothermal method that requires tens of hours to synthesize UHAPNWs, the microwave heating in this work can produce high-quality ultrathin UHAPNWs within 60 min, which can greatly enhance the reaction rate and efficiency, and shorten the reaction time.

Figure 5e shows the XRD patterns of the products synthesized by the microwave-assisted calcium oleate precursor hydrothermal method using creatine phosphate as the bio-phosphorus source at 180 °C for different times. The XRD patterns indicate that the as-prepared samples are composed of a single crystal phase of hexagonal hydroxyapatite (JCPDS No. 09-0432). This experimental result indicates that a single crystal phase of hydroxyapatite is formed in a short period of the microwave-assisted hydrothermal time (<15 min). With an increase in the reaction time, the crystallinity of the product is enhanced. Therefore, well-crystalline HAP nanowires have already formed within 30 min.

The experimental results show the microwave heating time has a great impact on the morphology and size of the product. It usually takes about 24 h or longer for the synthesis of UHAPNWs and other one-dimensional (1-D) hydroxyapatite nanomaterials by traditional heating methods [25,28,51,52,53,54,55,56,57,58,59], indicating the high efficiency and energy-saving advantage of the microwave heating method. The FTIR spectra (Figure 5f) show absorption peaks at 2931 cm^−1^ and 2854 cm^−1^, corresponding to the –CH_3_ and –CH_2_ groups, and absorption peaks at 1091 cm^−1^, 1027 cm^−1^, 603 cm^−1^, and 561 cm^−1^, belonging to the PO_4_^3−^ group [28,56], suggesting the formation of ultrathin UHAPNWs and the presence of organic components (oleate and creatine phosphate) on their surface. Ultrathin UHAPNWs with a uniform morphology, ultrasmall diameters, ultralong lengths, ultrahigh aspect ratios, and high crystallinity can be obtained when microwave hydrothermal heating is performed for a sufficient time, and 60 min is preferred.

**Figure 6:**
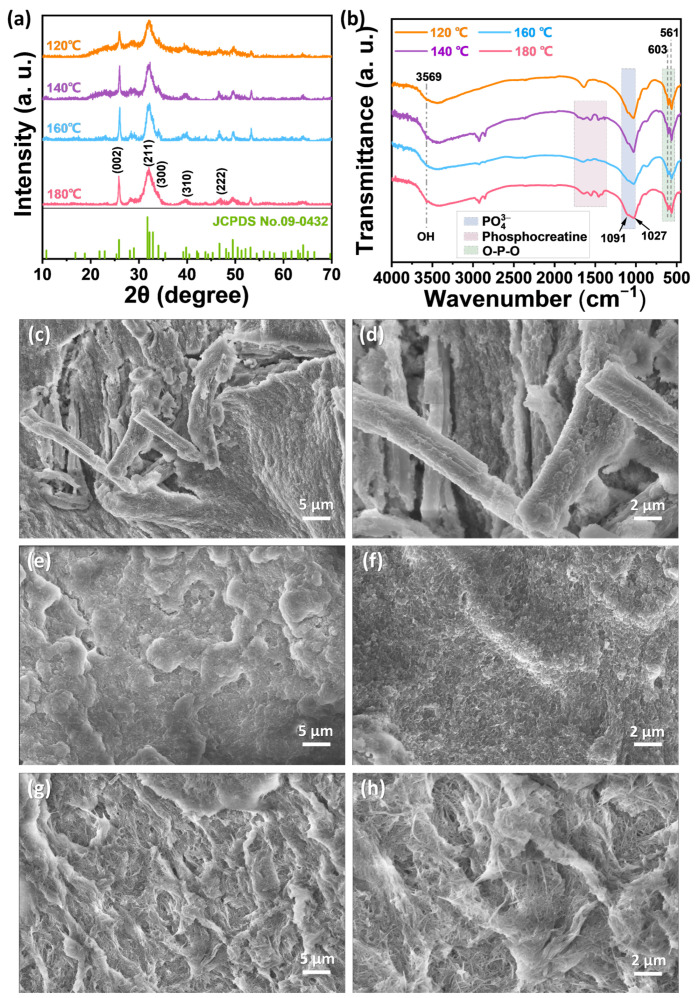
Characterization of the products obtained by the microwave-assisted calcium oleate precursor hydrothermal method using creatine phosphate as the bio-phosphorus source at different temperatures for 60 min. (**a**) XRD patterns; (**b**) FTIR spectra; (**c**–**h**) SEM images: (**c**,**d**) 120 °C, (**e**,**f**) 140 °C, and (**g**,**h**) 160 °C.

On this basis, we investigated the effect of microwave hydrothermal temperature on the formation of the product. As shown in Figure 6a, when the microwave hydrothermal temperature is 120 °C, the XRD pattern of the product shows a low crystallinity of hydroxyapatite and a broad diffraction peak from ~20°to ~40°, corresponding to amorphous calcium phosphate. That is, the product obtained by the microwave-assisted hydrothermal treatment at 120 °C for 60 min consists of a mixture of low-crystallinity hydroxyapatite and amorphous calcium phosphate. When the microwave hydrothermal temperature increases to 140 °C, the amount of the amorphous phase decreases significantly. When the microwave hydrothermal temperature is elevated to 160 °C or above, the product is composed of a single-phase crystalline hydroxyapatite with a hexagonal structure. The FTIR spectra of the products prepared by the microwave-assisted calcium oleate precursor hydrothermal method using creatine phosphate as the bio-phosphorus source at different temperatures for 60 min are shown in Figure 6b. The absorption peak at ~3569 cm^−1^ is ascribed to the –OH group of hydroxyapatite. The absorption peaks at 1027 cm^−1^ and 1091 cm^−1^ correspond to the stretching mode of the PO_4_^3−^ group, and the absorption peaks located at 603 cm^−1^ and 561 cm^−1^ correspond to the bending mode of the O–P–O of the PO_4_^3−^ group. Furthermore, the absorption peaks from 1300 to 1700 cm^−1^ originate from the presence of the groups from phosphocreatine, indicating that the phosphocreatine molecules are adsorbed on the surface of the products.

The experimental results indicate that the microwave hydrothermal temperature has a great effect on the morphology of the product (Figure 6c–h). When the microwave hydrothermal temperature is 120 °C or 140 °C, irregular structures are obtained, although their XRD patterns show the formation of the crystal phase of hexagonal hydroxyapatite. Thus, UHAPNWs cannot be prepared at low reaction temperatures by this method. From the crystallographic perspective, the oriented growth of hydroxyapatite into ultralong nanowires along its *c*-axis direction requires sufficiently high temperatures and pressures. As expected, when the reaction temperature is enhanced to 160 °C, UHAPNWs are formed without irregular structures, and the UHAPNWs become longer and thinner when the reaction temperature further increases to 180 °C. Therefore, an adequately high reaction temperature is essential for the formation of ultrathin UHAPNWs. In order to obtain ultrathin UHAPNWs, the minimum reaction temperature should be 180 °C.

**Figure 7:**
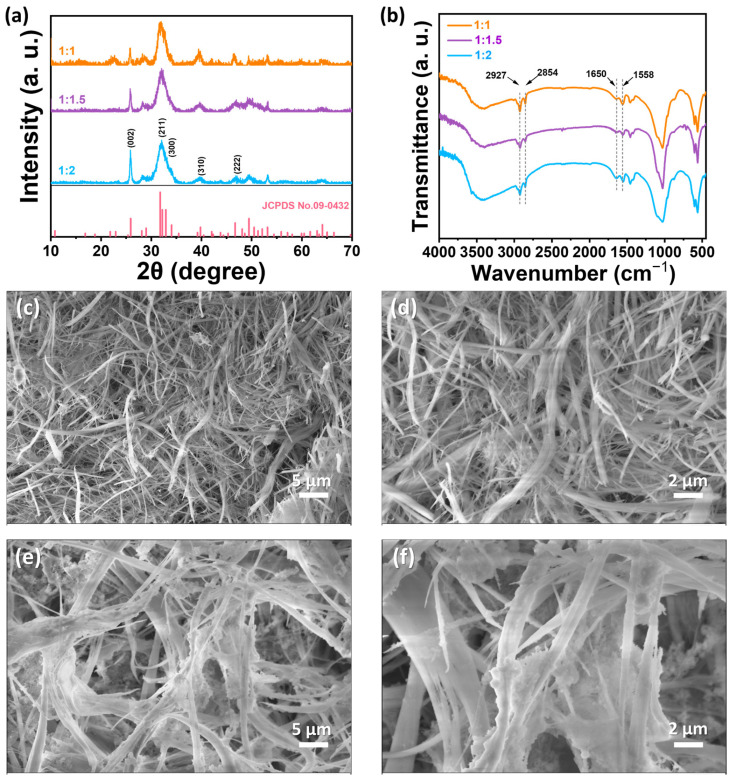
Characterization of the products prepared by the microwave-assisted calcium oleate precursor hydrothermal method using creatine phosphate as the bio-phosphorus source at different Ca/P molar ratios at 180 °C for 60 min. (**a**) XRD patterns; (**b**) FTIR spectra; (**c**–**f**) SEM images of the samples prepared at different Ca/P molar ratios: (**c**,**d**) 1:1 and (**e**,**f**) 1:1.5.

Furthermore, we investigated the influence of the Ca/P molar ratio of the reactants on the structure of the product (Figure 7). For the samples discussed above, we fixed the Ca/P molar ratio of the reactants at 1:2. Here, we modulated this ratio to 1:1.5 and 1:1 by varying the amount of creatine phosphate but fixing the amount of CaCl_2_. As shown in Figure 7a), the modulation of the Ca/P molar ratio of the reactants does not change the crystalline phase of the product, and all samples can be indexed to a single crystalline phase of hexagonal hydroxyapatite (JCPDS No. 09-0432). The FTIR spectra of the samples prepared at different Ca/P molar ratios of the reactants are shown in Figure 7b. The characteristic absorption peaks at 2927 cm^−1^ and 2854 cm^−1^, corresponding to the –CH_3_ and –CH_2_ groups, are observed in all FTIR spectra of the products. In addition, the characteristic absorption peaks at 1650 cm^−1^ and 1558 cm^−1^ are ascribed to the bending vibrations of –NH_2_ and C–N–H. These results suggest the presence of characteristic groups from phosphocreatine in the product.

The SEM investigation results reveal that the Ca/P molar ratio of the reactants also influences the morphology of the product. Although ultrathin UHAPNWs can be prepared by the modulation of Ca/P molar ratios ranging from 1:1 to 1:2, they exhibit a distinct size and uniformity. When the Ca/P molar ratio is 1:1, the as-prepared UHAPNWs show uneven diameters and lengths, and some thick HAP microfibers can be observed (Figure 7c,d). When the Ca/P molar ratio decreases to 1:1.5, the HAP nanowire bundles become thicker, with some particles. As discussed above, when the Ca/P molar ratio is 1:2, relatively uniform ultrathin UHAPNWs are synthesized. Therefore, hexagonal structured UHAPNWs can be obtained at different molar ratios of calcium and phosphorus sources of reactants, but ultrathin UHAPNWs with uniform sizes and higher aspect ratios are obtained when the Ca/P molar ratio is 1:2.

Therefore, the reaction time, reaction temperature, and Ca/P molar ratio of the reactants are important parameters to modulate the size and morphology of the product. We can conclude that the optimized parameters for the preparation of ultrathin UHAPNWs are as follows: adopting the microwave-assisted calcium oleate precursor hydrothermal method using creatine phosphate as the bio-phosphorus source heated at 180 °C for 60 min with the Ca/P molar ratio of the reactants being 1:2.

### 2.3. The Formation Mechanism of Ultrathin UHAPNWs

**Figure 8:**
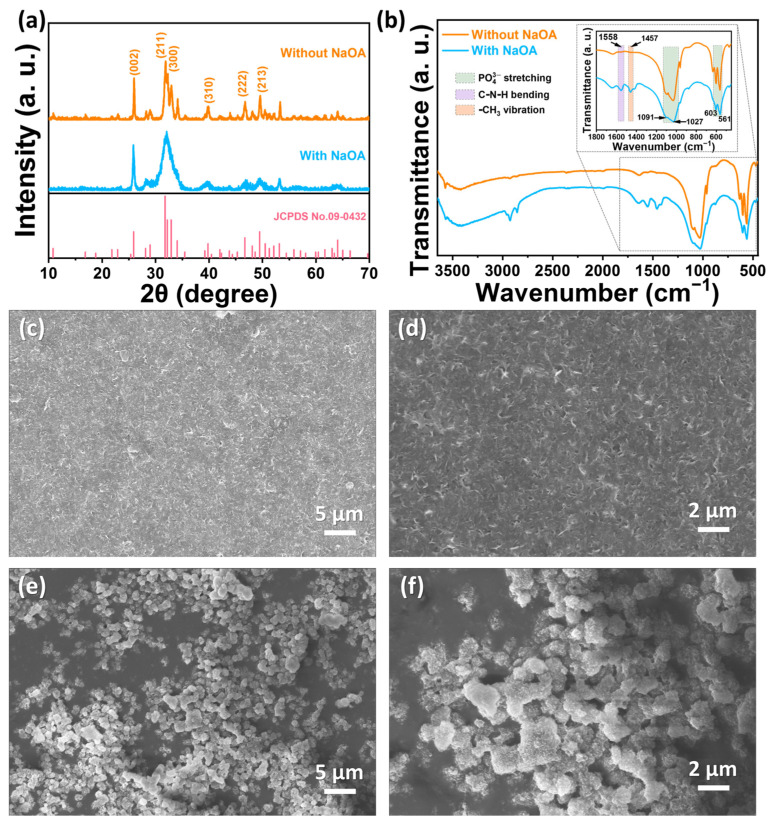
(**a**,**b**) XRD patterns (**a**) and FTIR spectra (**b**) of the products prepared by the microwave-assisted hydrothermal method using creatine phosphate as the bio-phosphorus source with or without sodium oleate at 180 °C for 60 min (Ca/P molar ratio 1:2). (**c**,**d**) SEM images of calcium oleate obtained by the reaction between sodium oleate and CaCl_2_ in an aqueous solution. (**e**,**f**) SEM images of the product synthesized by the microwave-assisted hydrothermal method using creatine phosphate as the bio-phosphorus source in the absence of sodium oleate at 180 °C for 60 min (Ca/P molar ratio 1:2).

The oleate plays an important role in the formation of ultrathin UHAPNWs. As shown in Figure 8a, although sodium oleate is absent from the reaction system, the XRD pattern of the obtained product is also indexed to a single crystal phase of hexagonal hydroxyapatite (JCPDS No. 09-0432). This is consistent with the results obtained with the synthetic system using sodium oleate. Therefore, sodium oleate does not alter the crystal phase of the product in this method. It is noted that the product obtained without sodium oleate shows a higher degree of crystallinity than ultrathin UHAPNWs prepared in the presence of sodium oleate. This may be attributed to the fact that ultrathin UHAPNWs possess ultrasmall diameters but extraordinarily long lengths, leading to the significant disarrangement of surface atoms. Similarly, as shown in Figure 8b, the FTIR spectra of the products obtained by the microwave-assisted hydrothermal method using creatine phosphate as the bio-phosphorus source both with and without sodium oleate exhibit the characteristic absorption peaks of PO_4_^3–^ located at 1091 cm^–1^, 1027 cm^–1^, 603 cm^–1^, and 561 cm^–1^, suggesting the formation of hydroxyapatite. However, the FTIR spectra of the ultrathin UHAPNWs prepared in the presence of sodium oleate show more pronounced C–N–H and –CH_3_ absorption peaks at 1558 cm^–1^ and 1457 cm^–1^, respectively, compared with those of the product prepared without sodium oleate. These peaks originate from creatine phosphate, suggesting that the interaction between oleate and creatine phosphate can better facilitate the in situ modification of the surface of ultrathin UHAPNWs with creatine phosphate molecules.

As shown in Figure 8c,d, the calcium oleate precursor formed by the reaction between oleate groups and calcium ions consists of irregular particles. During the microwave heating process, the hydrolysis of creatine phosphate results in the release of PO_4_^3–^ ions, and PO_4_^3–^ ions react with calcium oleate to gradually form ultrathin UHAPNWs. However, without the addition of sodium oleate to the reaction system, no calcium oleate precursor is formed, lacking structural guidance for the growth of ultrathin UHAPNWs. Therefore, only severely aggregated HAP particles are obtained in the reaction system in the absence of the calcium oleate precursor, and their diameters range from hundreds of nanometers to several micrometers (Figure 8e,f). The chemical reactions associated with the formation of ultrathin UHAPNWs using the calcium oleate precursor and creatine phosphate as the bio-phosphorus source is described below.

Ca^2+^ + 2R-COO^−^ → Ca(R-COO)_2_

Phosphocreatine + H_2_O → Creatine + H_3_PO_4_

10Ca(R-COO)_2_ + 6H_3_PO_4_ + 2H_2_O → Ca_10_(PO_4_)_6_(OH)_2_ + 20R-COOH

where R = CH_3_(CH_2_)_7_CH = CH(CH_2_)_7_.

Phosphocreatine is unable to hydrolyze in an aqueous solution at room temperature without creatine kinase, and can only hydrolyze at high temperatures without creatine kinase. Thus, unlike the case of inorganic phosphates as the phosphorus source, phosphocreatine can prevent undesired premature chemical reactions between calcium oleate precursor and PO_4_^3–^ ions before the microwave-assisted hydrothermal treatment.

**Figure 9:**
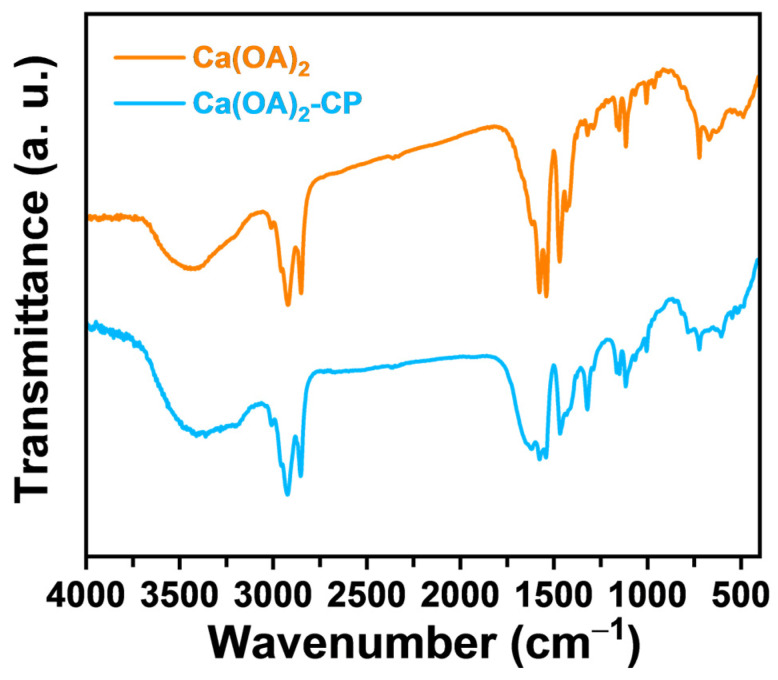
FTIR spectra of the calcium oleate (Ca(OA)_2_) precursor and the dried product (Ca(OA)_2_-CP) obtained by mixing the calcium oleate precursor with sodium creatine phosphate tetrahydrate in an aqueous solution at room temperature.

As shown in Figure 9, the FTIR spectrum of the dried product obtained by mixing the calcium oleate precursor and sodium creatine phosphate in aqueous solution (labelled as Ca(OA)_2_-CP) is consistent with that of the pure calcium oleate precursor (labelled as Ca(OA)_2_), indicating that the premature chemical reactions between calcium oleate precursor and sodium creatine phosphate did not occur. This can avoid the uncontrolled chemical reaction, nucleation, and crystal growth of the product.

In addition, microwave heating shows competitive superiorities such as environmental friendliness, high efficiency, and energy savings. Therefore, during the microwave-assisted hydrothermal treatment, the temperature of the reaction system is rapidly and uniformly elevated to the hydrolysis temperature of phosphocreatine, and the hydrolysis of phosphocreatine releases free PO_4_^3–^ ions in an aqueous solution to participate in the formation of ultrathin UHAPNWs. This can avoid the problem that calcium phosphate nuclei prematurely form and subsequently grow into byproducts during the lengthy heating process of the conventional hydrothermal treatment using inorganic phosphates as the phosphorus source, which results in an inhomogeneous morphology and size.

As expected, the experimental results verify our speculation that creatine phosphate is a rational phosphorus-containing biomolecule to control the formation of ultrathin UHAPNWs through the microwave-assisted calcium oleate precursor hydrothermal method. The advantages of creatine phosphate as a bio-phosphorus source for the synthesis of UHAPNWs are summarized as follows: (1) creatine phosphate is a biogenic and small biomolecule containing only one phosphate group in each molecule with the same breaking energy, which can provide PO_4_^3–^ ions for the formation of UHAPNWs by hydrolysis in an aqueous solution under hydrothermal conditions; (2) since the hydrolysis of creatine phosphate requires creatine kinase at room temperature, and can only hydrolyze at high temperatures in the absence of creatine kinase, this can prevent the undesired premature chemical reactions between calcium oleate precursor and PO_4_^3–^ ions before the microwave-assisted hydrothermal treatment; (3) the hydrolysis conditions for creatine phosphate to release PO_4_^3–^ ions are consistent with the hydrothermal treatment required for the formation of UHAPNWs; (4) creatine phosphate and its hydrolyzed product show high biocompatibility.

In our previous work, we chose ATP as the bio-phosphorus source for synthesizing UHAPNWs [32]. The chemical structure of creatine phosphate is very different from that of ATP, and thus their hydrolysis processes are different. Creatine phosphate and ATP cannot hydrolyze without enzymes at room temperature, and they can only hydrolyze at high temperatures in the absence of enzymes, but their hydrolysis behaviors are different at high temperatures under hydrothermal conditions. One ATP molecule contains three phosphate groups with different breaking energies, which gradually hydrolyze to form adenosine diphosphate, adenosine monophosphate, and adenosine under hydrothermal conditions, and the phosphate ions are released in an aqueous solution. In comparison, one creatine phosphate molecule contains only one phosphate group, and the same activated energy is needed for its hydrolysis. Therefore, creatine phosphate can hydrolyze to provide phosphate ions for the formation of UHAPNWs in a short period of time, which is beneficial for obtaining ultrathin UHAPNWs with a uniform morphology. Moreover, creatine phosphate, as a common drug for the treatment of hypertension and cardiovascular disease [60,61], is cheaper than ATP and thus is more promising to be used as the bio-phosphorus source for the synthesis of UHAPNWs with a reduced cost.

### 2.4. Ultrathin UHAPNWs as Drug Nanocarriers for Drug Delivery

**Figure 10:**
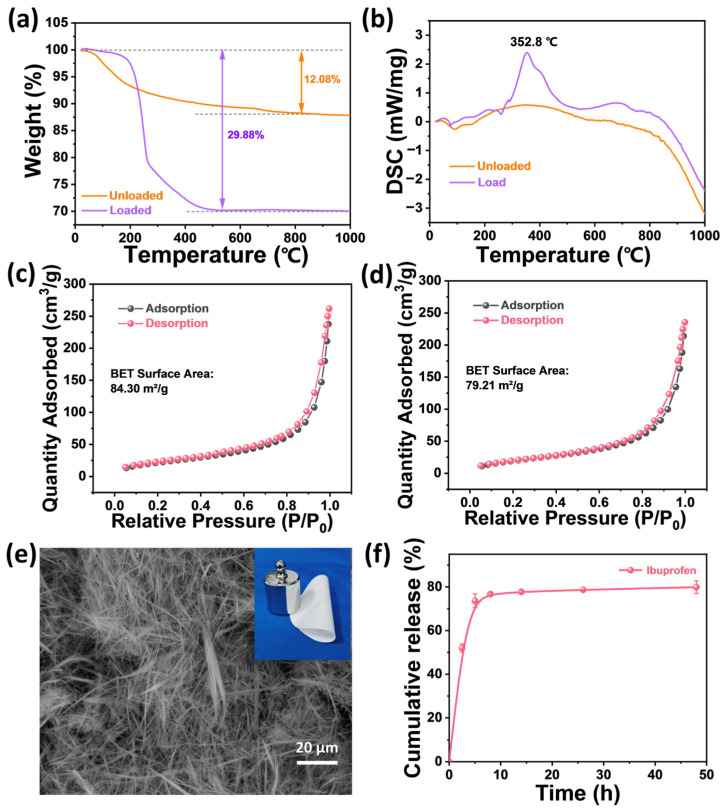
(**a**,**b**) Thermal analysis of the ultrathin UHAPNWs prepared by the microwave-assisted calcium oleate precursor hydrothermal method using creatine phosphate as the bio-phosphorus source at 180 °C for 60 min (Ca/P molar ratio 1:2) before and after loading ibuprofen: (**a**) TG curve; (**b**) DSC curve. (**c**,**d**) Nitrogen absorption/desorption curves of ultrathin UHAPNWs before (**c**) and after (**d**) ibuprofen loading. (**e**) SEM image of the ibuprofen-loaded ultrathin UHAPNW bio-paper. The inset is a digital image showing the high flexibility of the bio-paper. (**f**) Cumulative ibuprofen drug release profile of the ibuprofen-loaded ultrathin UHAPNW bio-paper in PBS (pH = 7.4, 37 °C).

Owing to their ultrasmall diameter, ultralong length, and ultrahigh aspect ratio, the as-prepared ultrathin UHAPNWs are the ideal nanocarrier for drug delivery. Herein, we investigated the drug loading performance of ultrathin UHAPNWs as the drug carrier using ibuprofen, a typical anti-inflammatory drug, as the model drug. The TG-DSC analysis was applied to calculate the drug loading capacity of ultrathin UHAPNWs. As shown in Figure 10a, the pure ultrathin UHAPNWs without ibuprofen loading exhibit a weight loss of about 12.08 wt.% from room temperature to 800 °C. This weight loss is ascribed to the evaporation of adsorbed water (5.32 wt.%), as well as the oxidative decomposition of creatine phosphate and residual oleate (6.76 wt.%). Thus, the organic content of UHAPNWs is calculated to be approximately 7.14%. The weight loss of ultrathin UHAPNWs loaded with ibuprofen is only 0.5 wt.% when it is heated from room temperature to 150 °C, mainly due to the evaporation of the adsorbed water. Compared with the TG curve of pure ultrathin UHAPNWs, the adsorbed water of ibuprofen-loaded UHAPNWs obviously decreases, which may be ascribed to the solvent exchange (water and hexane) during the ibuprofen loading process. When the temperature is further enhanced to 800 °C, the weight loss of ultrathin UHAPNWs loaded with ibuprofen reaches 29.88 wt.%. The increased weight loss results from the oxidative decomposition of ibuprofen. As evidence, there is a clear exothermic peak at 352.8 °C in the DSC curve of ibuprofen-loaded ultrathin UHAPNWs, whereas there is no obvious peak in the DSC curve of unloaded ultrathin UHAPNWs in the same temperature range (Figure 10b).

As the TG curve shows significant stepwise weight loss, it can be used to evaluate the ibuprofen loading. According to the thermogravimetric analysis results, the ibuprofen loading capacity (drug (*g*)/carrier (*g*)) in ultrathin UHAPNWs can be calculated using the following equation:

$$\mathrm{content}\;\left(\%\right)=1-\frac{m_{2}}{m_{1}}=\frac{m_{1}-m_{2}}{m_{1}}\times 100\%$$

$$\mathrm{Loading}\;\mathrm{capacity}\;(g/g)=\frac{(1-\frac{m_{2}}{m_{1}})}{\frac{m_{2}}{m_{1}}}=\frac{m_{1}-m_{2}}{m_{2}}$$

In the above equation, *m*_1_ and *m*_2_ are the percentages of the remaining weights of unloaded ultrathin UHAPNWs and ibuprofen-loaded ultrathin UHAPNWs, respectively, after being heated at a high temperature of 900 °C in the TG curves. According to this equation, the drug loading of ibuprofen in the ultrathin UHAPNW carrier is calculated as 0.33 g g^–1^, i.e., 0.33 g of ibuprofen can be loaded per gram of the ultrathin UHAPNW carrier, indicating the good drug loading capacity of the as-prepared ultrathin UHAPNWs.

In order to investigate the change in the ultrathin UHAPNWs before and after ibuprofen loading, the Brunauer–Emmett–Teller (BET) specific surface areas of the samples were measured. As shown in Figure 10c, the BET specific surface area of the pure ultrathin UHAPNWs without ibuprofen loading is measured to be about 84.30 m^2^ g^−1^, while after loading ibuprofen, the specific surface area is slightly reduced to 79.21 m^2^ g^−1^ (Figure 10d). In comparison with our previously reported ultralong UHAPNWs prepared by the calcium oleate precursor traditional hydrothermal/solvothermal method using the inorganic phosphate as the phosphorus source, the BET specific surface area of ultrathin UHAPNWs in this work is much higher because of the ultrasmall diameter, ultralong length, and ultrahigh aspect ratio. The high specific surface area of ultrathin UHAPNWs can provide plenty of active sites for drug loading, leading to a high drug loading capacity.

Furthermore, the ibuprofen-loaded ultrathin UHAPNWs can be interwoven into flexible and porous bio-paper. As shown in Figure 10e, the SEM image of the as-prepared bio-paper shows a continuous networked porous structure randomly interwoven with ibuprofen-loaded ultrathin UHAPNWs. This structure endows the bio-paper with high flexibility, which can be bent at large angles without noticeable damage (Figure 10e). The porous network structure provides a suitable microenvironment for cell adhesion, proliferation, and differentiation, and thus favors tissue regeneration [62,63,64]. In addition, the ibuprofen-loaded ultrathin UHAPNW bio-paper can sustainably release ibuprofen. As shown in Figure 10f, the ibuprofen drug is released at a suitable rate from the bio-paper in the first 12 h, which is beneficial for rapid anti-inflammatory pain relief. Then, the drug release rate decreases gradually.

**Figure 11:**
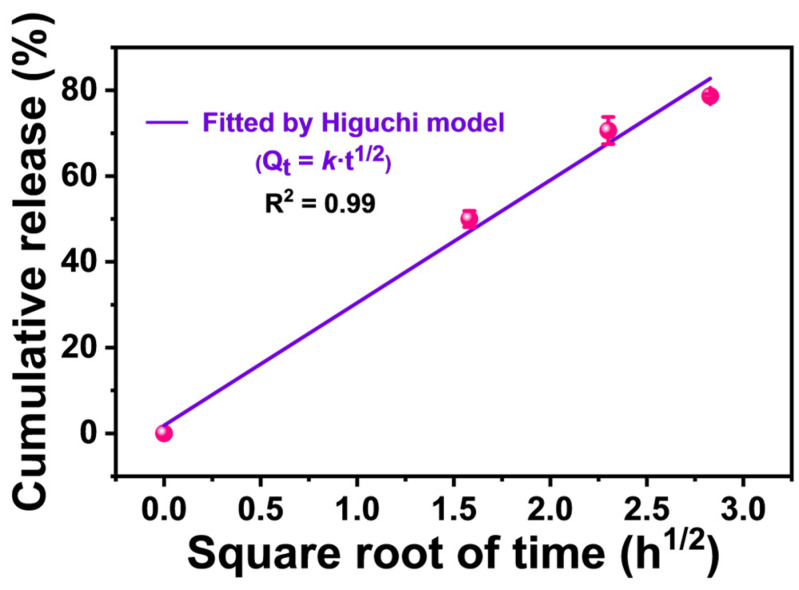
The linear relationship between the cumulative drug release percentage and the square root of the release time, which is consistent with the Higuchi model.

The drug release kinetics of ibuprofen from the ibuprofen-loaded ultrathin UHAPNW bio-paper can be described well by the Higuchi model, with a high coefficient of determination (R^2^) of 0.99 (Figure 11). According to this model, the cumulative amount of the released drug has a linear relationship with the square root of the release time

*Q*_t_ = *k*·*t*^1/2^

where *Q*_t_ is the amount of the drug released at time *t*, and *k* is the release rate constant for the Higuchi model [65,66]. This model is the common description for the diffusion-controlled release of a drug from insoluble carriers [67]. Therefore, as the ultrathin UHAPNWs exhibit a slow biodegradation rate in PBS, the drug release kinetics of ibuprofen from ultrathin UHAPNW bio-paper can be described as a process governed by the diffusion of drug molecules. It is expected that the as-prepared drug-loaded ultrathin UHAPNW bio-paper is promising for various biomedical applications such as anti-inflammatory, analgesic, wound healing, and bone defect repair.

## 3. Experimental

### 3.1. Materials and Chemicals

Calcium chloride (CaCl_2_) and hexane were purchased from Sinopharm Chemical Reagent Co., Ltd. (Shanghai, China). Sodium oleate and sodium creatine phosphate tetrahydrate were purchased from Aladdin Industrial Corporation. Ethanol was obtained from Shanghai Lingfeng Chemical Reagent Co., Ltd. (Shanghai, China). All chemicals were used as received without further purification. Deionized water was used in the related experiments.

### 3.2. Synthesis of Ultralong HAP Nanowires with Ultrathin Diameters

Ultralong HAP nanowires (UHAPNWs) with ultrathin diameters were synthesized by the microwave-assisted calcium oleate precursor hydrothermal method. Typically, CaCl_2_ (0.110 g) was dissolved in 5 mL deionized water. 2.634 g sodium oleate was dissolved in 15 mL hot deionized water, cooled, and then slowly added into the CaCl_2_ aqueous solution under magnetic stirring at room temperature. After 15 min of stirring, 5 mL of the aqueous solution containing 0.667 g sodium creatine phosphate tetrahydrate was added dropwise to the above mixture under magnetic stirring at room temperature. After that, the obtained aqueous suspension was transferred into an autoclave, sealed, and heated in a microwave oven (MDS-6, Sineo, Shanghai, China) for 60 min. The autoclave in this work was a modified polytetrafluoroethylene (TFM) cylindrical autoclave (total volume = 60 mL). Its inner diameter, outer diameter, and inner height were 3.0 cm, 3.8 cm, and 9.3 cm, respectively. It was fixed in a high-strength outer vessel. The microwave oven was a microwave hydrothermal/solvothermal synthesis system with a continuous heating mode, and the autoclave was continuously rotated during the microwave irradiation to ensure relatively uniform heating. After microwave heating, the autoclave was cooled down to room temperature, and the product was washed with ethanol and deionized water 3 times each. All samples were dried at 60 °C for 24 h before further use. Other samples were prepared by a similar procedures but with varying experimental parameters.

### 3.3. Material Characterization

X-ray powder diffraction (XRD) patterns were recorded by an X-ray diffractometer (D/max 2550 V, Rigaku, Tokyo, Japan) with Cu Kα radiation (λ = 1.54178 Å). Fourier transform infrared (FTIR) spectra were obtained with a FTIR spectrometer (FTIR-7600, Lambda Scientific, Edwardstown, Australia). Morphological observations of the samples were performed using scanning electron microscopy (SEM, Gemini 450, Zeiss, Oberkochen, Germany, and TM-3000, Hitachi, Tokyo, Japan) and transmission electron microscopy (TEM, JEM-2100F, JEOL, Tokyo, Japan). The elemental mapping of UHAPNWs was performed by SEM energy-dispersive X-ray spectroscopy (EDS, Ultim Extreme, Oxford Instrument, Oxford, UK) and SEM (Gemini 450, Zeiss, Oberkochen, Germany) at 10 kV. The samples were sputtered with gold for 15 s. The thermal properties of samples were tested by thermogravimetric (TG) analysis and differential scanning calorimetry (DSC) (STA 409/PC, Netzsch, Selb, Germany) at a heating rate of 10 °C·min^−1^ in flowing air. The Brunauer–Emmett–Teller (BET) specific surface area of the samples was measured by analyzing N_2_ adsorption/desorption using a surface area and pore size analyzer (Tristar II 3020, Micromeritics, Norcross, GA, USA). Before the measurement, the samples were outgassed at 80 °C for at least 4 h. The cumulative release of ibuprofen from the bio-paper of ibuprofen-loaded ultrathin UHAPNWs was assessed by soaking it in PBS at 37 °C under constant shaking (160 rpm) in a temperature-controlled shaker (HZQ-X 160, Peiying, Suzhou, China). At various intervals, 1 mL of the supernatant was withdrawn and analyzed by ultraviolet–visible (UV–vis) absorption spectroscopy (UV-2300II, Techcomp Instrument, Shanghai, China) to analyze its absorbance at a wavelength of 263 nm. Meanwhile, fresh PBS was added to replace the withdrawn volume to maintain a constant volume throughout the experiment.

### 3.4. Ibuprofen Loading

In a typical procedure of ibuprofen drug loading, ibuprofen was dissolved into hexane to form a solution with a concentration of 50 mg mL^–1^. Then, 50 mg of the as-prepared powder of ultrathin UHAPNWs was added into 20 mL of the above ibuprofen solution under magnetic stirring. It should be noted that the container was covered with a lid to avoid the evaporation of hexane. After being soaked for 4 h, the supernatant was removed, and the ibuprofen-loaded ultrathin UHAPNWs were collected, washed with clean hexane, and dried to obtain the ibuprofen-loaded ultrathin UHAPNW drug delivery system.

## 4. Conclusions

To sum up, we have developed the microwave-assisted calcium oleate precursor hydrothermal method for the rapid synthesis of ultrathin UHAPNWs using creatine phosphate as the bio-phosphorus source. This method has the advantages of environmental friendliness, efficient heating, a short reaction time, high efficiency, and energy savings. The controllable hydrolysis of phosphocreatine prevents the undesired premature formation of calcium phosphate nuclei, and microwave heating rapidly and uniformly elevates the temperature for phosphocreatine hydrolysis, which releases free PO_4_^3–^ ions for the formation of homogeneous ultrathin UHAPNWs. Ultrathin UHAPNWs with ultrathin diameters (only ~6 nm for a single nanowire), ultralong lengths (several hundred micrometers), and ultrahigh aspect ratios (>10,000) can be rapidly prepared within 60 min, shortening the synthesis time by about two orders of magnitude compared with the traditional hydrothermal method. Ultrathin UHAPNWs can self-assemble along their longitudinal direction to form nanowire bundles with a high BET specific surface area (84.30 m^2^ g^−1^). Moreover, the as-prepared ultrathin UHAPNWs show a high ibuprofen loading capacity (0.33 g drug per gram of nanocarrier). After loading ibuprofen, ultrathin UHAPNWs still exhibit a high specific surface area of 79.21 m^2^ g^−1^. The ibuprofen-loaded ultrathin UHAPNWs can be further interwoven into flexible and porous bio-paper, and it can continuously release the drug in phosphate-buffered saline, which is promising for various biomedical applications such as inflammatory treatment and accelerated tissue regeneration. In future research, the in vitro/in vivo biocompatibility of the as-prepared ultrathin UHAPNWs as drug nanocarriers should be investigated. Moreover, experiments can be performed to load other drugs in the ultrathin UHAPNWs drug nanocarriers for a variety of biomedical applications.

## Data Availability

The data presented in this study are available on reasonable request from the corresponding authors.
